# Developing an Australian utility value set for the Early Childhood Oral Health Impact Scale-4D (ECOHIS-4D) using a discrete choice experiment

**DOI:** 10.1007/s10198-022-01542-x

**Published:** 2022-11-17

**Authors:** Ruvini M. Hettiarachchi, Peter Arrow, Sameera Senanayake, Hannah Carter, David Brain, Richard Norman, Utsana Tonmukayawul, Lisa Jamieson, Sanjeewa Kularatna

**Affiliations:** 1 Australian Centre for Health Services Innovation (AusHSI) and Centre for Healthcare Transformation, School of Public Health and Social Work, Queensland University of Technology (QUT), Brisbane, Queensland Australia; 2 Australian Research Centre for Population Oral Health, Adelaide Health and Medical Sciences, University of Adelaide, Adelaide, Australia; 3 School of Population Health, Curtin University, Perth, Australia; 4 Deakin Health Economics, Institute for Health Transformation, Faculty of Health, Deakin University, Victoria, Australia; 5 Health Department Western Australia, Dental Health Services, Western Australia, Australia; 6 Dental School, University of Western Australia, Perth, Australia

**Keywords:** Preference based, Quality-adjusted life years, Children, Health state valuations, Discrete choice experiments, Economic evaluation, Oral health, Early childhood, Pediatric, C35

## Abstract

**Purpose:**

Preference-based quality of life measures (PBMs) are used to generate quality-adjusted life years (QALYs) in economic evaluations. A PBM consists of (1) a health state classification system and (2) a utility value set that allows the instrument responses to be converted to QALYs. A new, oral health-specific classification system, the Early Childhood Oral Health Impact Scale-4D (ECOHIS-4D) has recently been developed. The aim of this study was to generate an Australian utility value set for the ECOHIS-4D.

**Methods:**

A discrete choice experiment with duration (DCE_TTO_) was used as the preference elicitation technique. An online survey was administered to a representative sample of Australian adults over 18 years. Respondents were given 14 choice tasks (10 tasks from the DCE design of 50 choice sets blocked into five blocks, 2 practice tasks, a repeated and a dominant task). Data were analyzed using the conditional logit model.

**Results:**

A total of 1201 respondents from the Australian general population completed the survey. Of them, 69% (*n* = 829) perceived their oral health status to be good, very good, or excellent. The estimated coefficients from the conditional logit models were in the expected directions and were statistically significant (*p* < 0.001). The utility values for health states defined by the ECOHIS-4D ranged from 0.0376 to 1.0000.

**Conclusions:**

This newly developed utility value set will enable the calculation of utility values for economic evaluations of interventions related to oral diseases such as dental caries among young children. This will facilitate more effective resource allocation for oral health services.

**Supplementary Information:**

The online version contains supplementary material available at 10.1007/s10198-022-01542-x.

## Introduction

Oral diseases are among the most prevalent childhood diseases globally. Over 530 million children suffer from dental caries in their primary teeth, the most prevalent childhood oral disease [1]. Often, children's poor oral health leads to negative consequences such as problems with eating, speaking, learning, and self-esteem [2]. Children with poor oral health miss more school days and show poorer performance in school grades than children with optimal oral health [3]. The 2012–2014 Australian National Child Oral Health Survey revealed that more than 25% of Australian children aged 5–10 years had at least one untreated carious tooth in the primary dentition and an average of 1.5 decayed, missing, or filled teeth [4, 5]. Further, more than 20% of 5- to 14-year-old Australian children had gingivitis [5].

Oral treatments are costly and impose a significant burden on health care systems and individuals. Around 5% of the total health expenditure of most high-income countries is directed for dental treatment, whereas the provision of dental care is beyond the capacity of most low- and middle-income countries [1]. The total dental expenditure in the USA was $101 billion in 2016 [6], while Australia spent AU$10.5 billion for overall dental services in 2017–2018 [4].

Economic evaluations provide an important framework to prioritize health interventions in resource-scarce settings. They assist health-care decision-making by providing information on health interventions with the best value for money [7]. Quality-adjusted life year (QALYs) is a single measure that combines both the length and quality of life [8] and the preferred outcome measure in economic evaluations, which allow comparisons between health programs in different disease areas [7]. Preference-based quality of life measures (PBMs) are commonly used to calculate the quality of life component of QALYs [9]. These measures can be generic or disease specific [9]. The widely used generic PBMs for children are the Child Health Utility nine-dimension (CHU-9D), EuroQol Five-Dimension Youth Questionnaire (EQ-5D-Y), Health Utility Index 2 (HUI2), 16 Dimensions (16D), and Assessment of Quality of Life-6 Dimension (AQoL-6D) [10]. Generic child-specific PBMs such as the CHU-9D have been used in previous oral health research [11, 12]. However, evidence suggests that these generic measures may not be sensitive to important changes in oral health outcomes [11, 12]. Most available pediatric oral health-related quality of life measures are non-preference based. Therefore, they cannot be used to calculate utility values to derive QALYs for economic evaluations.

To address the need for a pediatric oral health-specific preference-based measure, we developed a new oral health-specific classification system, the Early Childhood Oral Health Impact Scale-4D (ECOHIS-4D) [13]. ECOHIS-4D is a proxy-reported PBM targeted at children under 7 years. This was the first stage in developing a PBM for this age group which could be used to estimate QALYs in future economic evaluations. The second and final stage involves a health state valuation study to assign a utility value set to the health states described by the ECOHIS-4D.

Several methods have been used to derive preference weights for health states and to develop a utility algorithm for health states defined by a classification system [14]. Discrete choice experiments (DCE) are a preference elicitation method that has been used widely in such studies [14]. DCE methods are becoming increasingly prominent in health state valuation research [15] as they are compatible with online survey platforms, providing a time- and resource-efficient approach. In this study, we report on a DCE study to generate a preference-based utility value set for the health states defined by the ECOHIS-4D.

## Methods

### Construction of discrete choice experimental design

Discrete choice tasks produce utility values on a latent scale (i.e., preferences are measured in unanchored utility without units) [16]. Hence, it is important to anchor the utilities generated from the DCE design into the 'full health = 1' to 'dead = 0' scale to calculate health state utility values [16]. Several methods have been applied to anchor utility values [15]. The inclusion of an additional duration attribute to the choice task is the most commonly adopted anchoring approach [15]. The duration attribute provides information on how respondents trade-off time in a health state followed by  death, thus allowing the anchoring of preferences to the 0–1 utility-scale [16]. In this study, we conducted a DCE with an additional attribute of duration (often called DCE_TTO_,) as the preference elicitation method, using the methods described by Bansback et al. [17].

**Fig. 1:**
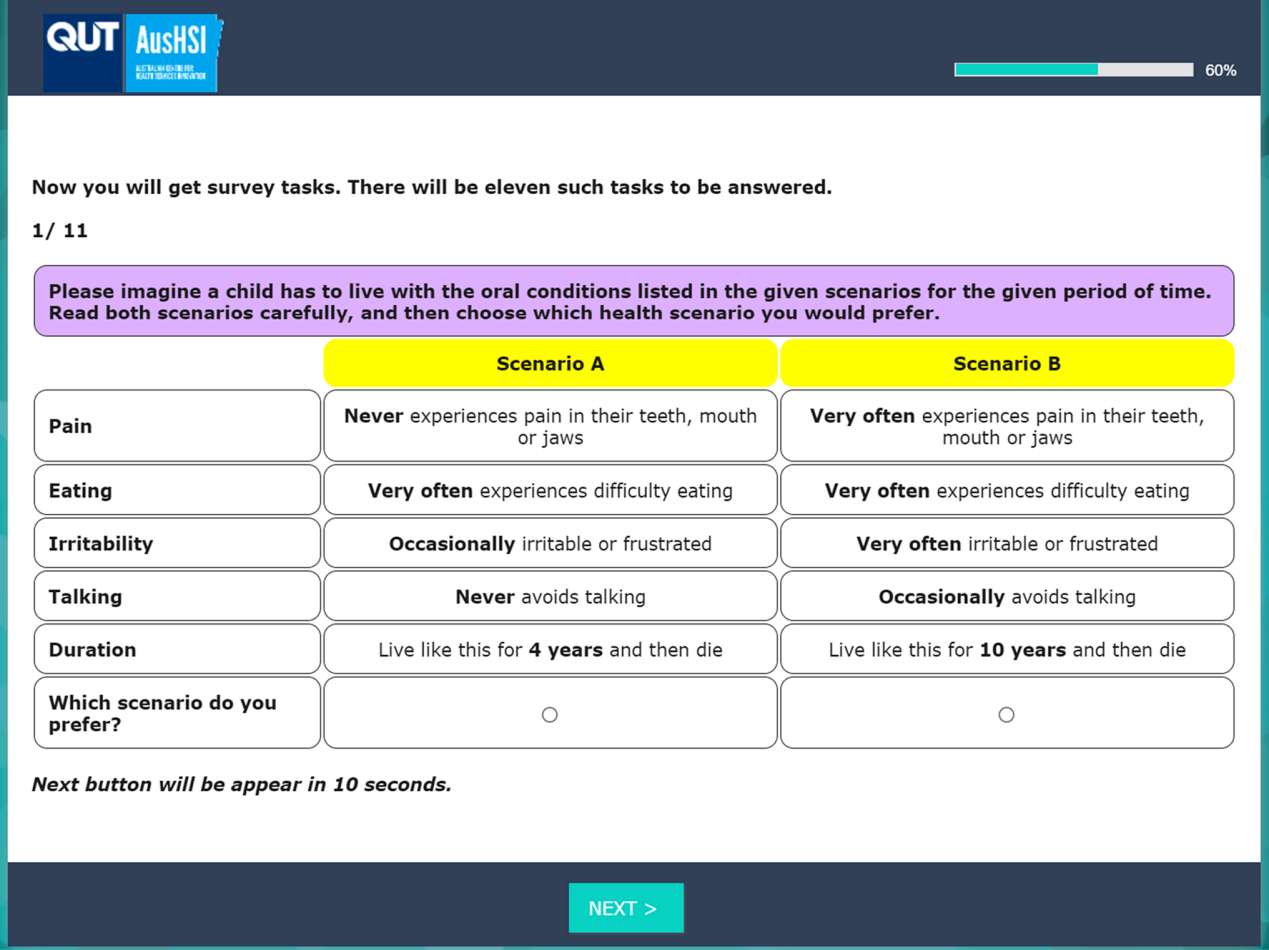
Example DCE choice task

The ECOHIS-4D classification system (Table 1) consists of four dimensions: pain, eating, irritability, and talking. Each dimension has three ordinal frequency levels (never, occasionally, and very often). An additional duration attribute consisted of four levels (6 months, 4 years, 7 years, and 10 years) to evaluate individuals' preferences concerning survival durations. As such, the DCE_TTO_ choice sets were designed to contain five attributes in total, describing one level from each of the ECOHIS-4D dimensions and one duration level. An example of a DCE_TTO_ choice task is shown in Fig. 1.

**Table 1:** ECOHIS-4D classification system

Dimension	Level	Description
Pain	1	Your child never experiences pain in the teeth, mouth, or jaws
	2	Your child occasionally experiences pain in the teeth, mouth, or jaws
	3	Your child very often experiences pain in the teeth, mouth, or jaws
Eating	1	Your child never experiences difficulty eating
	2	Your child occasionally experiences difficulty eating
	3	Your child very often experiences difficulty eating
Irritability	1	Your child is never irritable or frustrated
	2	Your child is occasionally irritable or frustrated
	3	Your child is very often irritable or frustrated
Talking	1	Your child never avoids talking
	2	Your child occasionally avoids talking
	3	Your child very often avoids talking

The choice tasks were designed using Ngene. The experimental design determines the total number of health states to be included in the valuation study and the combinations of health states to be valued by each respondent. The ECOHIS-4D instrument (four dimensions and three levels in each) and the duration attribute (four levels) could define 324 (3^4^ × 4^1^) health states and 52,326 [324 × (324 − 1)/2] possible pairwise combinations [28]. It was not practical to value all possible combinations with full factorial design. Therefore, a D-efficient design was generated to select an optimal subset containing 50 pairwise choice sets of these health states that would maximize the efficiency of the survey design [18] . 

### Respondents

The ECOHIS-4D is a parent-proxy measure for younger children [13]. As this questionnaire would be completed by adults (typically primary caregivers) we were interested in understanding how adults would perceive and value the health states described in the survey. Therefore, for elicitation of utility values in the DCE study, we determined that a sample of the general Australian adult population would be used [19, 20]. The inclusion criteria for the DCE were adults aged 18 + years irrespective of parenthood status. However, information on parenthood status, including the number of children currently under 12 years old, was captured within the demographic information within the survey. Survey respondents were recruited with the help of an existing Australian online panel, PureProfile (www.pureprofile.com). Quotas were set for age, gender, and geographic area during recruitment for the online survey to ensure the sample was an approximation of the Australian population.

### Pilot studies

The study included two pilot studies prior to the main survey. The main aim of the first pilot (*n* = 101) was to obtain priors and to identify any issues related to the wording and understandability of choice set tasks, attributes, and their levels, as well as the functioning of the survey instrument. In the absence of any published priors, the Ngene design of the first pilot study was developed using very small priors (i.e., 0.00001) with correct direction signs; negative or positive to indicate the direction of preference for each coefficient. Each respondent in the first pilot was given just five-choice tasks from the design to reduce the respondents’ burden.

Priors estimated from the first pilot study were used to develop a second D-efficient DCE design to be used for the second pilot study and the main survey. The minimum number of choice tasks “*s*” was determined by the number of parameters “*k*” to be estimated, therefore for the “*j*” number of alternatives, the minimum number of choice tasks was estimated using the published formula: (*j* − 1)*s* ≥ *k* [21]. There were nine coefficients to be estimated for the ECOHIS-4D: one duration coefficient and separate coefficients for levels 2 and 3 of each of the four ECOHIS-4D attributes (base levels were excluded as they were denoted by zero). This required nine choice tasks as the minimum number of choice tasks with two alternatives. After the first pilot study, the number of choice tasks from the DCE design was increased from five-choice tasks to ten choice tasks per respondent. Thus, the second D-efficient DCE pilot design included the design codes based on the priors obtained from the first pilot study and the full design was divided into five blocks (versions) of the survey, with ten choice sets per block. In addition to these ten choice tasks per respondent, an additional two tasks; a repeated choice task and a dominant choice task, were also included to assess the internal reliability and consistency of responses. The choice tasks commenced with two practice tasks for respondents to become familiar with the choice tasks procedure. Hence, each participant was given 14 choice sets in total for the second pilot study.

The second pilot study was conducted among another 116 respondents to identify any survey deficiencies with the new DCE design. These responses were used to check for the ordering and statistical significance of the coefficients. There were no changes made to the survey following the second pilot; hence the survey design was continued as the main survey and the data from the second pilot sample was also included in the main survey analysis.

### Sample size

The sample size for a DCE study is based on the characteristics of the study design, such as the number of attributes, the size of the population, and the statistical power required of the model derived [21]. Based on the s-error estimated from the second D-efficient design, the sample size estimation for the main survey was 1200. Therefore, we set our recruitment target at 1200 members of the general population.

### Data collection

A web-based survey was administered to a sample of the Australian general population during December 2021 to January 2022. PureProfile sent an initial invitation along with the participant information sheet and link to the consent screen and the survey. In the first section, respondents were provided with an introduction to the study and were invited to provide consent to continue the survey. Demographic data including gender, age, education, marital status, and employment were collected. If participants had children, they were then asked about their age and they completed additional questions related to their child’s oral health-related quality of life using the ECOHIS-4D questionnaire. The next section of the survey consisted of DCE_TTO_ tasks. A detailed description of the choice tasks was included, and respondents were also given information and instructions on how to complete the DCE_TTO_ with two practice tasks provided. At the end of the DCE_TTO_ task, respondents were asked to rate their difficulty completing this exercise.

Ethical approval for the study was obtained from the Queensland University of Technology Human Research Ethics Committee (LR 2021-4456-5557).

### Statistical analysis

Data were analyzed using Stata 16 software [22]. We used a conditional logit model under a random utility framework to analyze DCE_TTO_ data. A random utility framework assumes that the respondents choose the alternative that maximizes their utility. The utility function consists of a vector of observable attributes and a random error term (Eq. 1) [23].

1 $${U}_{ijk}={V}_{ijk}+{\varepsilon }_{ijk}$$

${V}_{ijk}$ is the fixed utility that individual *i* would get from choosing option *k* in the choice set *j. *${\varepsilon }_{ijk}$ is the unobservable random error term.

### Model specification

As the main objective of this health state valuation was to generate a utility value set for health states defined by the ECOHIS-4D, the specific utility function for the DCE_TTO_ responses were modeled using the approach developed and described by Bansback et al. [17]. These values were then anchored onto the 0–1 (death to full health) scale (required to generate QALYs).

The estimated coefficients were then anchored onto the 0–1 scale to derive utility values corresponding to each health state. The sample mean DCE_TTO_ value for the state *x*_*ij*_ can be calculated from the coefficients of the conditional logit model [17]. These estimates implied the average amount of life expectancy that respondents are willing to trade-off for an improvement in the given health dimension (Eq. 2).

2 $$U_{ijk} = \frac{{\beta^{\prime}_{ij} {\varvec{x}}_{ij} \times t_{ij} }}{{\beta_{0} t_{ij} }}$$

${U}_{ij}$ is the utility individual *i* would get from choosing option *k* in the choice set *j*, ${\beta }_{0}$ is an estimate of the utility associated with the life years attribute *t*, $\beta^{\prime}_{ij}$ is an estimate of the utility associated with the level of each dimension in *x*_*ijk*_ for each life year attribute *t*, ${{\varvec{x}}}_{ijk}$ is a vector of eight binary dummy variables $({x}_{ijk}^{12},{x}_{ijk}^{13},\dots ,{x}_{ijk}^{43})$, representing each level of four health attributes.

Therefore, utility decrements for each level away from level 1 (base level) in each of the four ECOHIS-4D attributes were estimated by dividing each of the $\beta^{\prime}$ terms by ${\beta }_{0}.$ 95% confidence intervals around these ratios were estimated using the STATA wtp command [22].

### Sensitivity analysis and assessing preference heterogeneity

Conditional logit models are widely used in choice modeling, hence our choice of this method for the primary analysis [23]. However, the data were also explored using mixed logit models in subsequent analyses to evaluate preference heterogeneity (Eq. 3). Compared to Eq. (2), in addition to the *β*_0_ and *β*′_1_ representing mean preference in the population, this also includes *γ*_*i*_ and *η*_*i*_ as the individual variation from mean preference [20].

3 $$U_{ijk} = (\beta_{0} + \gamma_{i} ) t_{ijk} + (\beta^{\prime}_{1} + \eta_{i} ) {\varvec{x}}_{ijk} \times t_{ijk} + \varepsilon_{ij} $$

Additional sensitivity analyses were also conducted that estimated conditional logit model models that: (1) excluded respondents who did not answer the dominant choice task correctly; (2) excluded those who gave different answers for the repeated question.

## Results

Initial invitations were sent to 1861 respondents, and 1201 respondents completed the online survey (*n* = 366 were screened out due to quota sampling and *n* = 302 did not complete the survey). The basic characteristics of the sample are summarized in Table 2. The respondents generally approximated the Australian general population in relation to the balance of age, gender, and current state of residence (Table 2). However, the sample had a higher educational level than the Australian general population. The mean age of the sample was 47.52 years. Around 70% rated their oral health as good, very good, or excellent and 64% of the respondents stated that their teeth and mouth bothered them a little or not at all in their everyday life (Supplementary Table 1). Nearly 28% (*n* = 331) had children under 12 years of age. Of these, around 95% reported that their children never or occasionally had pain, difficulty eating, or avoided talking due to conditions in their teeth, mouth, or jaw. Almost all (87%) reported that their children were never or occasionally irritable or frustrated due to their teeth, mouth, or jaws (Supplementary Table 1).

**Table 2:** Comparison of socio-demographic characteristics of the sample with the Australian general population (*n* = 1201)

Characteristic	Sample number	%	Population value^a^	*p*-value^#^
Age, mean (SD)	47.52 (17.68)		39.09	
Min–max	18–91 years			
Age (years)				
18–24 years	124	10.32	12.05	0.6854
25–34 years	238	19.82	19.24,	
35–44 years	209	17.4	17.10	
45–54 years	202	16.82	16.28	
55–64 years	179	14.9	14.88	
65–74 years	146	12.16	11.57	
75 + years	103	8.58	8.88	
Gender				
Male	587	48.88	49.3	0.9566
Female	608	50.62	50.7	
Non-binary	5	0.42		
Prefer not to say	1	0.08		
State of residence				
New South Wales	385	32.06	31.80	0.9746
Victoria	307	25.56	26.08	
Queensland	245	20.4	20.12	
South Australia	84	6.99	6.89	
Western Australia	129	10.74	10.36	
Australia Capital Territory	18	1.5	1.68	
Tasmania	25	2.08	2.10	
Northern Territory	8	0.67	0.96	
Highest level of education, *n* (%)				
Grade 10	160	13.32	15.4^b^	< 0.001
Grade 12	189	15.74	18.2	
Certificate II–IV	215	17.9	17.8	
Diploma	152	12.66	13.5^c^	
Bachelor’s degree	356	29.64	19.8	
Postgraduate degree (master’s/PhD)	114	9.49	8.5	
Other	15	1.25	6.8^d^	
Marital status*, *n* (%)				
Single	361	30.06	35.03	< 0.001
Married/de facto	658	54.79	48.07	
Divorced/widowed	158	13.16	13.7	
Other	24	2.00	3.2	
Current employment status, *n* (%)				
Full-time employment	508	42.3		
Part-time employment	222	18.48		
Unemployed	134	11.16		
Pension	107	8.91		
Retired	230	19.15		

The highest level of education (Education and Work, Australia, Australian Bureau of Statistics, May 2021 from https://www.abs.gov.au/)

^#^ The Chi-squared goodness-of-fit test was used to compare observed frequencies with population proportions

^a^Australian age and sex distribution (Australian Bureau of statistics, June 2020 from https://www.abs.gov.au/

^b^Australian population data for grade 10 and 11

^c^Australian population data for advanced/graduate diploma

^d^Australian population data for highest education level below grade 10

Of the 1201 respondents, 6.32% (*n* = 76) did not correctly complete the dominant task, and 23.31% (*n* = 280) did not correctly complete the repeated task. The whole sample (*n* = 1201) was included in the base case analysis, and sensitivity analysis was performed by excluding the respondents who incorrectly completed the dominant and/or repeated tasks. The median duration of the entire survey was 7.1 min, and the mean completion time was 20.16 min (± standard deviation 104.60; range 3.19–2148.22) (Supplementary Table 2). The range for the completion time was extremely wide as the respondents had the opportunity to save their responses and complete the survey at their convenience. Around 25% stated that selecting between two options was easy or very easy, 26% reported that it was not difficult, 30% of the respondents found it difficult, and less than 20% reported that selecting preferences from the DCE choice tasks was very difficult (Supplementary Table 2).

### DCE model analysis

DCE choice tasks were analyzed using the conditional logit model as specified in Eqs. (1) and (2). The estimated coefficients were in the expected direction and logically consistent with the correct sign (a negative sign in the attribute levels indicate utility decrements compared with the base level). All coefficients were statistically significant (*p* < 0.001) (Table 3). The mixed logit model was also estimated to assess the preference heterogeneity, and the coefficients were statistically significant, in the expected direction, and consistent. For both models, the duration coefficient was positive and statistically significant, which implied the utility increased with higher life expectancy (Table 3).

**Table 3:** Estimated coefficients for the model comparisons

	Conditional logit (*n* = 1201)		Mixlogit (*n* = 1201)		Excluding respondents with the incorrect dominant task (*n* = 1125)		Excluding respondents with incorrect repeat tasks (*n* = 921)	
Estimated mean								
	Coefficient	SE	Coefficient	SE	Coefficient	SE	Coefficient	SE
Duration	0.42498*	0.01651	0.62556*	0.02374	0.44990*	0.01708	0.45319*	0.01932
Pain × duration								
2	− 0.04755*	0.00467	− 0.07674*	0.00652	− 0.04970*	0.00491	− 0.05360*	0.00544
3	− 0.15251*	0.00741	− 0.22764*	0.01165	− 0.15990*	0.00772	− 0.16127*	0.00872
Eating × duration								
2	− 0.01790*	0.00473	− 0.04454*	0.00677	− 0.01899*	0.00491	− 0.01680*	0.00541
3	− 0.09725*	0.00631	− 0.15240*	0.00985	− 0.10110*	0.00660	− 0.09823*	0.00727
Irritability × duration								
2	− 0.04411*	0.00480	− 0.05422*	0.00694	− 0.04621*	0.00503	− 0.05311*	0.00563
3	− 0.08685*	0.00489	− 0.12119*	0.00717	− 0.09058*	0.00508	− 0.09479*	0.00567
Talking × duration								
2	− 0.02063*	0.00460	− 0.03649*	0.00642	− 0.02169*	0.00466	− 0.02120*	0.00538
3	− 0.07238*	0.00460	− 0.11829*	0.00733	− 0.07461*	0.00471	− 0.07518*	0.00537
Estimated standard distribution								
			Coefficient	SE				
Pain × duration								
2			0.07093	0.01096				
3			0.18129	0.01007				
Eating × duration								
2			0.00166	0.02126				
3			0.12034	0.00880				
Irritability × duration								
2			− 0.06007	0.01376				
3			0.07044	0.01085				
Talking × duration								
2			0.01438	0.02208				
3			0.09194	0.00993				
Estimation statistics								
Log likelihood	− 7653.3449		− 7420.2924		− 7105.3156		− 5805.4867	
AIC	15,324.69		14,874.58		14,228.65		11,628.97	
BIC	15,397.47		15,012.06		14,300.84		11,699.36	

*AIC* Akaike information criterion, *BIC* Bayesian information criterion, *SE* standard error

*Significant coefficients at *p* < 0.001 level

**Fig. 2:**
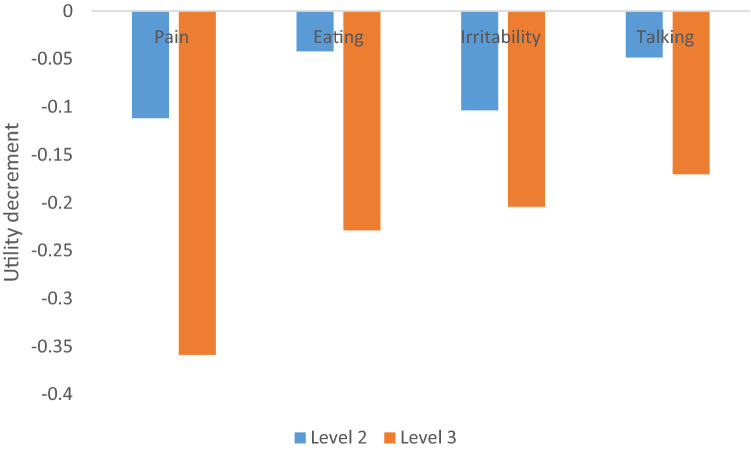
Utility decrement in each attribute

For both the conditional logit model and the mixlogit models, estimated coefficients were consistent with the correct sign and statistically significant. Therefore, being the most parsimonious model, the conditional logit model was selected to anchor the 0–1 QALY scale. The utility decrement for each attribute level of ECOHIS-4D as derived in the conditional logit model is shown in Table 4 and Fig. 2. Of the ECOHIS-4D attributes, "pain" generated the highest utility decrement followed by "eating", "irritability" and "talking". This indicates pain is the most impactful factor when selecting preferences between two health states. The attribute "irritability" showed the smallest difference in the strength of preference between the second level and third level, highlighting that there is less difference between "occasionally irritable or frustrated" and "very often irritable or frustrated" when compared to relative differences in levels across the other ECOHIS domains.

**Table 4:** Anchored values for each attribute level based on the conditional logit model (*n* = 1201)

	Utility decrement^#^	95% CI
Pain		
2	− 0.11189	− 0.09188 to − 0.13190
3	− 0.35886	− 0.33658 to − 0.38114
Eating		
2	− 0.04213	− 0.02162 to − 0.06263
3	− 0.22883	− 0.20866 to − 0.24901
Irritability		
2	− 0.10379	− 0.08330 to − 0.12427
3	− 0.20436	− 0.18455 to − 0.22418
Talking		
2	− 0.04855	− 0.02779 to − 0.06932
3	− 0.17032	− 0.14988 to − 0.19075

^#^Anchored value = estimated coefficient for each level in dimension/duration coefficient

The utility value for each health state can be calculated using the utility decrements provided in Table 4. For example, the utility decrements for the worst level of pain, eating, irritability, and talking attributes are 0.35886, 0.22883, 0.20436, and 0.17032 respectively. Therefore, the utility value for the worst health state (3333) would be,

Health state 3333 = 1 − (0.35886 + 0.22883 + 0.20436 + 0.17032) = 0.03763.

The utility values for health states defined by the ECOHIS-4D classification system range from 0.0376 (worst health state 3333) to 1.0000 (full health state 1111). The utility algorithm based on the utility decrements provided in Table 4 can be used to calculate utility values when data is collected using the ECOHIS 13 item oral health-related quality of life measure. R codes and STATA codes to derive these utility values from the data set of the ECOHIS 13 item scale are provided in Supplementary files 3.

### Sensitivity analysis

Two other models were estimated, excluding respondents with incorrect dominant tasks and incorrect repeat tasks (Table 3) using the conditional logit model. For both models, estimated coefficients were in a logical order with the correct signs and were statistically significant. The subsample analysis excluding respondents with incorrect repeat or dominant tasks did not meaningfully improve the model. Therefore, these were not considered in the final model to estimate the anchored coefficients to derive utility values.

## Discussion

We developed a utility algorithm and utility value set to generate preference weights for the health states defined by the new oral health-specific classification system, the ECOHIS-4D. This utility value set will enable the calculation of QALYs using the new oral health-specific PBM, ECOHIS-4D, to be used in economic evaluations of pediatric oral health interventions in the cost-utility analysis framework. In addition, we have produced a utility algorithm that can be used to convert responses to the ECOHIS 13 item oral health-related quality of life measure to utility values for deriving QALYs.

There is evidence that condition-specific measures are more sensitive to capturing the health-related quality of life changes due to interventions targeting specific diseases [12]. The availability of the ECOHIS-4D classification system and the utility value set will enable the accurate calculation of utility values in economic evaluations of pediatric oral health interventions. ECOHIS-4D is a proxy-reported PBM targeted at young children, specifically those who are under 7 years. Most currently available pediatric oral health-related quality of life measures are non-preference based; hence they cannot be used to calculate utility values to derive QALYs for economic evaluations. There are two pediatric oral health-specific PBMs currently available, both targeted at older children. The Caries impacts and experiences Questionnaire for Children (CARIES-QC) is an oral health-specific PBM targeted at 5–16 years old children [24], whereas the target group for the Dental Caries Utility Index (DCUI) is 12–17 years [25]. Evidence suggests that early childhood is the best time to improve oral health, with good oral health in childhood being the strongest predictor of good oral health in adulthood [26]. Young children experience a higher prevalence of dental caries, with higher relative treatment needs and associated costs, and are therefore commonly targeted for oral health interventions [9]. The ECOHIS-4D is an important tool that can be used to inform future economic evaluations of oral health interventions among these younger children.

The utility value set we report on here was developed based on the recommended guidelines [27, 28] and informed by the methods used in the previous DCE studies to estimate utility value sets for preference-based classification systems [17]. The DCE experimental choice sets were generated using a D-efficient design, the most commonly used design when constructing DCE experiments for PBM utility elicitation [28]. Our use of pilot studies to obtain priors for the main DCE choice design was based on the evidence that D-efficient designs generated using informative priors are more statistically efficient than those created with non-informative priors or zero priors [28].

Among the four ECOHIS-4D attributes, "Pain" generated the highest utility decrement, indicating pain as the most concerning factor when trading-off attributes between health states. This observation is consistent with what has been reported in other oral health valuation studies. The health state valuation of the Dental Caries Utility Index (DCUI) also reported "Pain/discomfort" with the highest utility decrement compared to the other attributes in DCUI [29]. Acharya et al. [30] reported that respondents who had "pain" as the main complaint had higher standard gamble utility scores, indicating a higher willingness to accept risks to achieve better oral health. Studies using time trade-off and visual analog scale methods to value dental health states reported "painful decayed tooth" as having the lowest median utility values indicating less willingness to trade-off [31].

The utility values for the ECOHIS-4D ranged from 0.0376 (worst health state) to 1.0000 (full health state). The worst health state valued for the ECOHIS-4D is comparatively lower than that of other generic and oral health-specific pediatric PBMs. For example, the worst health state for the Child Health Utility Index 9D, a generic pediatric PBM for 7–11 years, is valued at 0.3368 by the UK adult general population [32]. The worst health state of DCUI (3333) was valued at 0.1681 [29]. The worst state of the ECOHIS-4D was valued higher than the CARIES-QC another oral health-specific PBM worst health state (CARIES-QC 33333 valued at − 0.326 in adolescent value set) [24]. However, the techniques used in these health state valuations, as well as the attributes and levels contained within these PBMs, differ from those in the ECOHIS-4D making direct comparisons difficult.

There is ongoing debate as to who is best placed to value health states for pediatric PBMs. A common view is that, as taxpayers, the general adult population’s preferences are important for deciding which treatments should be funded through public health systems [33]. Previous studies have used adult general population samples to generate value sets for both generic [34] as well as condition specific [20] pediatric PBMs. It is also common to use adult samples to value pediatric health states across conditions that are common to both pediatric and adult populations [35]. In Australia, dental caries is ranked among the top ten causes of non-fatal disease burden among children as well as adults 25–44 years of age [36]. Hence, adults are often familiar with the signs and symptoms of common oral diseases affecting childhood. Recent research encourages children to value health states defined by the pediatric PBMs as they would be experiencing the health states quite differently from adults [37]. However, for proxy measures such as ECOHIS-4D with a target group of very young children, it is not feasible for the target cohort to complete the questionnaire or value the health states [38]. In addition to the question of who should value child health states, is a broader question of whether a patient or a general population sample should be considered for health state valuation of condition specific PBMs. There is mixed evidence as to whether the general population vs patient samples produce significantly different utility values [39–41]. There is some evidence that patients may place higher values for disease-related health states due to a natural adaptation to these states [42]. These may in turn results in an underestimation of the cost-effectiveness of new treatments [42]. Conversely, while the general population may not have direct experience of particular health states, they may provide unbiased judgment, hence providing justice as decision makers. For this reason, health technology assessment bodies for publicly funded health systems generally recommend using general population samples for health state valuation informing system level decisions on resource allocation [42]. Nevertheless, we suggest future studies should consider ECOHIS-4D health state valuation using parents of young children with oral diseases to evaluate the differences between health state values of the general population and a patient-proxy sample.

Valuation perspective is another important factor to be considered in health state valuation studies. The health state valuation of Health Utilities Index 2 (HUI2), a generic pediatric PBM, asked the respondent to imagine that they were a child aged 10 years [10, 32]. However, it was reported that this presented difficulties as some respondents tried to remember when they were 10 years old, while others considered imaginary 10-year-old children or their own adulthood experience during the valuation interviews [32]. The perspective used in valuing health states in the CHU-9D was that of adults, and the respondents were asked to imagine that they were in the described health state. CARIES-QC has used both adult and adolescent perspectives in health state valuation [24]. Authors of the CHU-9D valuation suggested that the relatively high value placed on the worst health state could be due to the adult perspective in valuing the health states, without knowing that it was related to children [32]. In the ECOHIS-4D health state valuation reported here, we have used a valuation approach and perspective informed by insights from this previous body of research. During the valuation tasks, respondents were asked to imagine a hypothetical child when choosing between the health scenarios provided. This could be a contributing factor to the relatively low utility value placed on the worst health state. Childhood oral diseases are generally non-fatal. However, our findings indicate that respondents had a strong preference for not wanting a child to live in the worst health states, thus would have resulted in lower utility values for severe attribute levels. Less than 20% of respondents reported that choosing between the two given health scenarios as being very difficult, indicating that this approach is feasible to be used in pediatric PBMs health state valuations. We recommend that future studies continue to extend knowledge in this area by comparing the impact of adopting different valuation perspectives (e.g., an adult experiencing the health state, an imaginary child in the health state, or a respondent remembering being a child when valuing the health state).

Although ECOHIS-4D is a proxy measure with caregivers being the respondent, health state valuation in this study was not confined to parents. Instead, the sample included adults over 18 years of age, irrespective of parenthood status. This is consistent with the approach of assigning general community preferences to health states [20, 32]. However, within our sample approximately 28% were adults of children aged 12 years and younger. This may have impacted the preferences of this subgroup of parents if they were more likely to relate the scenarios to their own lived experiences. To overcome any potential distress associated with choosing between scenarios that included a limited duration of child survival, the survey included an introductory page prior to the DCE choice tasks with very simple language explaining the nature of the DCE choice tasks. It also emphasized that it was highly unlikely that any oral health conditions described will impact a child's survival and explained the rationale for including limited survival time as an attribute within the choice sets. In future valuation studies of parent proxy-based PBMs, we recommend that comparisons are made between a parent-only sample versus a general adult population sample.

### Limitations

This study has some limitations to note. The survey was conducted using a convenience sample obtained via an online platform, which meant the sample was not representative of the general Australian population. The sample broadly reflected the general adult population in terms of age, sex, and state of residence. However, the sample is not representative in terms of other characteristics such as education level. This could be because the certain group of people for example people with good computer literacy are normally registered for the online survey panels. This is a common limitation among other online studies as well. An additional limitation is that it was not possible to understand how completely and accurately respondents understood the survey questions when using an online platform.

## Conclusion

The newly developed utility value set for ECOHIS-4D will enable the calculation of utility values to be used in economic evaluations of pediatric oral health interventions. This will be facilitated using the oral health-specific preference-based measure ECOHIS-4D for oral health interventions among younger children. A utility algorithm for the ECOHIS 13 item quality of life instrument is also available. This may ultimately lead to more effective and efficient resource allocation and planning of oral health services for younger children.

### Supplementary Information

Below is the link to the electronic supplementary material.

Supplementary file1 (TXT 8 KB)

Supplementary file2 (DOCX 20 KB)
