# Supplementary material for: Developing an Australian utility value set for the Early Childhood Oral Health Impact Scale-4D (ECOHIS-4D) using a discrete choice experiment
Source: Eur J Health Econ. 2022 Nov 17;24(8):1285–96. doi: 10.1007/s10198-022-01542-x (PMC10533628; doi:10.1007/s10198-022-01542-x)
Supplement: Supplementary file 2 — Supplementary file2 (DOCX 20 KB) [file 10198_2022_1542_MOESM2_ESM.docx]

**Supplementary Table 1: Perceived oral health status of the respondents and their children**

| **Characteristic** | **Number** | **%** |  |
| --- | --- | --- | --- |
| Reported oral health status |  |  |  |
| Very poor | 17 | 1.42 |  |
| Poor | 87 | 7.24 |  |
| Fair | 268 | 22.31 |  |
| Good | 418 | 34.80 |  |
| Very good | 303 | 25.23 |  |
| Excellent | 108 | 8.99 |  |
|  |  |  |  |
| How teeth and mouth bother everyday life |  |  |  |
| Not at all | 384 | 31.97 |  |
| A little | 386 | 32.14 |  |
| Some | 248 | 20.65 |  |
| A lot | 117 | 9.74 |  |
| Very much | 66 | 5.5 |  |
|  |  |  |  |
| Have children |  |  |  |
| Yes | 331 | 27.56 |  |
| No | 870 | 72.44 |  |
|  |  |  |  |
| Reported oral health status of their children using ECOHIS-4D (n=331) | | | |
|  | **Never** | **Occasionally** | **Very often** |
| Pain in teeth, mouth, or jaws n (%) | 148 (44.71) | 162 (48.94) | 21 (6.34) |
| Difficulty eating due to condition of teeth, mouth, or jaws n (%) | 194 (58.61) | 114 (34.44) | 23 (6.95) |
| Irritable or frustrated due to condition of teeth, mouth, or jaws n (%) | 78 (23.56) | 210 (63.44) | 43 (12.99) |
| Avoids talking teeth, mouth, or jaws n (%) | 209 (63.14) | 108 (32.63) | 14 (**4.23)** |

**Supplementary Table 2: Survey** **characteristics**

| Length of Interview (Minutes) | Total sample (n=1201) | Excluding respondents did more than 10 hours  (n= 1192) | Excluding respondents did more than 1 hour  (n= 1169) |
| --- | --- | --- | --- |
| Mean (SD) | 20.16 (104.60) | 12.17 (27.40) | 9.38 (7.68) |
| Median | 7.1 | 7.04 | 6.97 |
| Min-max | 3.19-2148.22 | 3.19-538.9 | 3.19-57.83 |
|  |  |  |  |
| Difficulty in completing DCE tasks, n (%) |  |  |  |
| Very difficult | 219 | 18.23 |  |
| Difficult | 374 | 31.14 |  |
| Not difficult | 314 | 26.14 |  |
| Easy | 193 | 16.07 |  |
| Very Easy | 101 | 8.41 |  |
